# Spatial positional relationship between macular superficial vessel density and ganglion cell-inner plexiform layer thickness in primary angle closure glaucoma

**DOI:** 10.1007/s10792-021-02005-7

**Published:** 2021-08-15

**Authors:** Yongdong Lin, Di Ma, Hongxi Wang, Shirong Chen, Weihao Cai, Anlin Zhang, Mingzhi Zhang

**Affiliations:** Joint Shantou International Eye Center of Shantou University, The Chinese University of Hong Kong, Dong xia Road, Shantou, 515041 Guangdong Province People’s Republic of China

**Keywords:** Optical coherence tomography angiography, Macular superficial vessel density, Primary angle closure glaucoma, Macular ganglion cell-inner plexiform layer thickness, Foveal avascular zone

## Abstract

**Purpose:**

To evaluate the spatial relationship between macular superficial vessel density (SVD) and macular ganglion cell-inner plexiform layer (GCIPL) thickness in primary angle closure glaucoma (PACG), and to investigate diagnostic abilities of macular SVD and foveal avascular zone (FAZ) parameters.

**Methods:**

This was a cross-sectional study on 38 PACG patients (38 eyes) and 25 healthy subjects (25 eyes). Macular region was imaged using a 1050-nm-wavelength swept-source optical coherence tomography (OCT) angiography (OCTA) system (DRI OCT Triton, TOPCON). Vessel density of the macular region was quantified by ImageJ software. The peripapillary retinal nerve fiber layer (pRNFL) thicknesses and macular GCIPL thickness were obtained by swept-source OCT. Pearson correlation analysis was used to evaluate the spatial positional relationship between macular SVD and macular GCIPL thickness. At the same time, the correlation between macular SVD and pRNFL thickness was evaluated. Areas under the receiver operating characteristics curves (AUCs) of OCT, OCTA and FAZ measurement metrics were calculated to assess the diagnostic ability for glaucoma.

**Results:**

Macular GCIPL thickness had a moderate correlation with the macular SVD in the inferonasal sector (r = 0.426, *P* = 0.008). In addition, there was a strong correlation between inferonasal sector of macular vessel density and 5,6,7,8 clock-hour regions of the pRNFL thicknesses (all r > 0.5). Inferoinferior sector of macular SVD and 6,7 clock-hour regions of pRNFL thicknesses also had strong correlation (all r > 0.5). The AUCs of macular SVD ranged between 0.61 (superonasal sector) and 0.76 (inferoinferior sector). The FAZ circularity index showed the highest diagnostic power (AUC = 0.94;95% CI, 0.85–0.99), followed by superotemporal sector of macular GCIPL thicknesses (0.93;95% CI,0.83–0.98).

**Conclusions:**

Sector of macular SVD not only had a spatial positional correlation with corresponding macular GCIPL thickness, but also with clock-hour regional pRNFL thicknesses in PACG eyes. FAZ circulation index might be a useful diagnostic parameter.

## Introduction

Primary angle closure glaucoma (PACG) is more likely to cause blindness than primary open-angle glaucoma (POAG), of which more than 80% are in Asia [1–3]. Recently, the study of PACG by optical coherence tomography angiography (OCTA) showed that peripapillary vessel density decreased [4–7]. But studies on PACG using OCTA were mainly on the optic disk area, less on the macular region.

More than 50% of ganglion cell bodies are located in the macular area, where the thicknesses of ganglion cells are 8–10 layers [8, 9]. Ganglion cells are supplied by the macular superficial vascular complex [10]. Previous studies using OCTA found that macular vessel density decreased and showed fairly diagnostic power in PACG eyes [11, 12]. Hood et al. [13]' s study demonstrated that inferior region of the macula was more prone to damage in glaucoma. Perhaps the diagnostic ability of macular vessel density in the inferior area is higher. However, the diagnostic ability of macular vessel density in each sector remained unclear in PACG. The foveal avascular zone (FAZ) is surrounded by interconnected capillary beds in the center of the macula [14]. The FAZ is highly susceptible to ischemia. Diabetic retinopathy and retinal vein occlusion can lead to the expansion of FAZ area [15, 16]. Previous study reported that FAZ parameters in POAG had good diagnostic ability by using OCTA [17]. As far as we know, there is only one study reported that the FAZ circularity index showed high diagnostic power in PACG patients [12]. But no studies investigated the FAZ's diagnostic ability for PACG when compared with traditional optical coherence tomography (OCT) parameters.

In a previous study, Richter et al. [18] used OCTA to study macular vessel density in POAG. They found that the vessel density of ganglion cell-inner plexiform layer (GCIPL) decreased, and only inferior sector GCIPL thickness was associated with corresponding vessel density. Kim et al. [19] studied the macular superficial vessel density (SVD) of early normal-tension glaucoma (NTG) and suspected glaucoma, and found that sectoral macular SVD were highly correlated with the corresponding macular GCIPL and peripapillary retinal nerve fiber layer (pRNFL) thicknesses. However, little is known about relationship between macular SVD and GCIPL thickness in PACG.

Therefore, in the present study, we evaluated the spatial relationship between macular SVD and GCIPL thickness in PACG, and to investigate diagnostic abilities of macular SVD and FAZ parameters.

## Methods

### Patients

This was a cross-sectional study. We told each subject the content of the study. The study protocol was approved by the Ethics Committee of Joint Shantou International Eye Center (JSIEC) of Shantou University and the Chinese University of Hong Kong (Shantou city, China). The study followed the tenets of the Declaration of Helsinki. Written informed consent was obtained from all subjects. Our study included 38 PACG patients who consecutively attended the Glaucoma Clinic at the JSIEC from September 2018 to June 2020. At the same time, 25 age- and sex-matched healthy patients were recruited.

All subjects underwent a detailed medical history, best-corrected visual acuity (BCVA), intraocular pressure (IOP) measurement, axial length measurement by OA-2000 (Tomey GmbH, Nagoya, Japan), fundus examination, swept-source OCT and OCTA (DRI OCT Triton, TOPCON) examination. We used the same DRI OCT instrument to perform OCT and OCTA scans. Glaucomatous patients underwent standard visual field (VF) examination by the static automated white-on-white threshold 24–2 SITA standard strategy (Humphrey Field Analyzer II; Carl Zeiss Meditec). This study only included reliable visual field examination results (false-negative errors < 15%, false-positive errors < 15%, and fixation loss < 20%).

PACG was defined by occludable anterior chamber angles in 2 or more quadrants on gonioscopy with goniosynechiae. Patients with PACG had optic nerve head changes characteristic of glaucoma (focal or diffuse neuroretinal rim thinning, localized notching, or nerve fiber layer defects) with correlating reliable visual field defects [20]. Our study did not include acute angle closure glaucoma. Inclusion criteria for healthy eyes had normal anterior chamber, open-angle and fundus in clinical examination by experts. Healthy eyes also had intraocular pressure ≤ 21 mmmhg and no family history of glaucoma. Exclusion criteria for all subjects were: 1. age: < 18 years old. 2. diopter ≥ 6.0 D (sphere) and or 3.0 D (cylinder). 3. previous eye surgery or ocular laser surgery and other eye diseases.

### OCTA and OCT imaging acquisition

All subjects were examined using the macular 6 × 6 mm scanning protocol (DRI OCT Triton; Topcon Corporation, Tokyo, Japan). Topcon OCTA instrument uses a wavelength of 1050 nm with A-scan rate of 100,000 scans per second [21]. The instrument produces maps using OCTA ratio analyses (OCTARA), which is an amplitude-decorrelation ratio-based algorithm [22]. The system automatically divided the macula into four layers, and the selected layer was superficial retinal capillary plexus (SCP). The SCP is defined as from the inner border of the retinal nerve fiber layer to 15.6 μm from the boundary between the inner plexiform layer and the inner nuclear layer. We checked and filtered images quality after each scan. Images with significant motion artifacts, or poor image clarity were excluded.

Macular OCT scan was performed immediately after OCTA scan. The system automatically gained a macular GCIPL thickness annulus with an inner diameter of 1 mm and an outer diameter of 6 mm. Then the annulus was automatically divided into 6 equal sectors (SN, superonasal; SS, superosuperior; ST, superotemporal; IT, inferotemporal; II, inferoinferior; IN, inferonasal).

### OCTA imaging processing

**Fig. 1:**
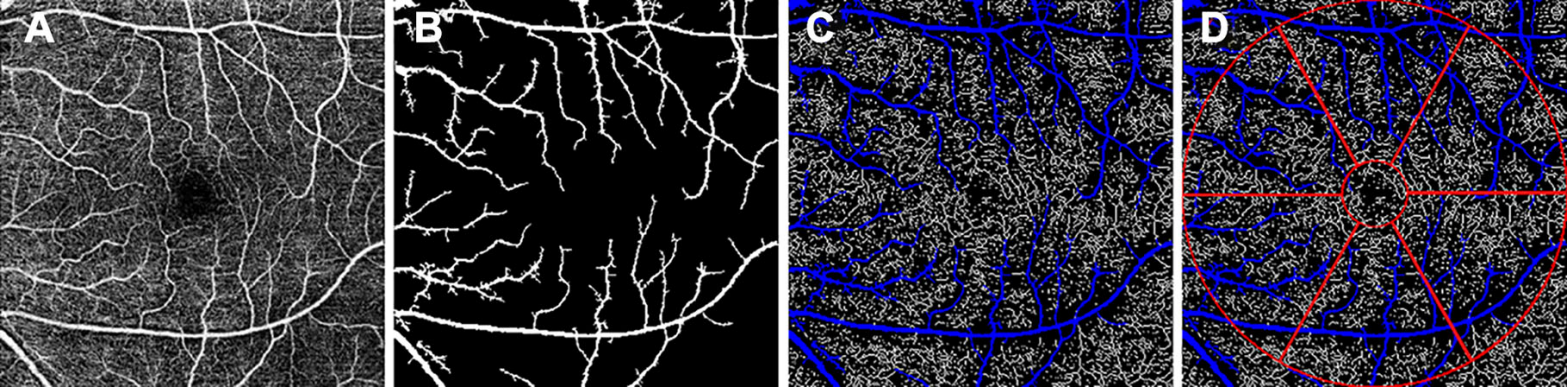
OCTA image processing steps. **A** Image imported into the image J software and denoising process. **B** Main vessel image obtained by adjustable threshold tool. **C** After removing main vessels in skeletonised image and then whole 6 × 6 mm macular superficial vessel density was calculated **D** Six equal sectors vessel density values of annulus centered on the fovea

All OCTA images were analyzed using Image J software (National Institutes of Health, Bethesda, MD). OCTA image processing steps are presented in Fig. 1. First, we used non-local mean (NLM) denoising filter to reduce the background noise (Fig. 1A). Second, we applied the adjustable threshold tool. The tool automatically set lower and upper thresholds (130–255, respectively, in this study). After the threshold tool was applied, the main vessel related pixels were obtained (Fig. 1B). Third, we used Niblack as adaptive local thresholding algorithm to binarize the gray image, and then the image was skeletonised [23]. Shoji et al. [24] proved that the reproducibility of analyzing macular vessel density using Niblack algorithm was good. After removing areas occupied by main blood vessels from the skeletonized image, the vessel density was calculated (Fig. 1C). The macular SVD was defined as the total vessel length per unit area [19]. The macular area was measured in an annulus with an inner diameter of 1 mm and outer diameter of 6 mm centered on the fovea and the annular region was divided into 6 equal sectors (Fig. 1D).

### Measurement of FAZ parameters

Researcher used imageJ software to manually mark FAZ of SCP images without knowing the clinical information of each participant, and FAZ metrics (area and perimeter) were then collected. The manual measurement of FAZ was repeated two times by two researchers (WC and AZ). The final metrics of FAZ were taken as the average of the results measured by two researchers to minimize the effect of inter-rater variation. Circularity index was measured using the following formula [25, 26]:

$${\text{Circularity }}{\text{index}} = 4\pi \text{area/perimeter}^{2}$$

To assess the intraobserver and interobserver reproducibilities of the measurements of FAZ metrics, intraobserver correlation coefficients (ICCs) with 95% confidence intervals were calculated.

### Statistical analysis

We used SPSS (ver. 22.0; SPSS Inc, Chicago, IL) analysis software and MedCalc (ver.15.2.2, Mariakerke, Belgium) for all statistical analysis. The Shapiro–Wilk test was performed to evaluate the normal distribution of continuous variable data. We compared the parameters between glaucomatous eyes and healthy eyes by independent t test, nonparametric Mann–Whitney test and chi-square test. Pearson correlation analysis was used to assess the correlation between the macular SVD and the corresponding macular GCIPL thickness, clock-hour RNFL thicknesses in glaucomatous eyes. We evaluated the relevant level by coefficient value (r), as follows: 0.10–0.29 = weak, 0.30–0.49 = moderate and over 0.50 = strong [19]. We calculated and compared the areas under the receiver operating characteristics curves (AUCs) of OCT, OCTA and FAZ measurement metrics to evaluate the diagnostic ability for glaucoma. *P* value of < 0.05 was considered to be of statistical significance.

## Results

**Fig. 2:**
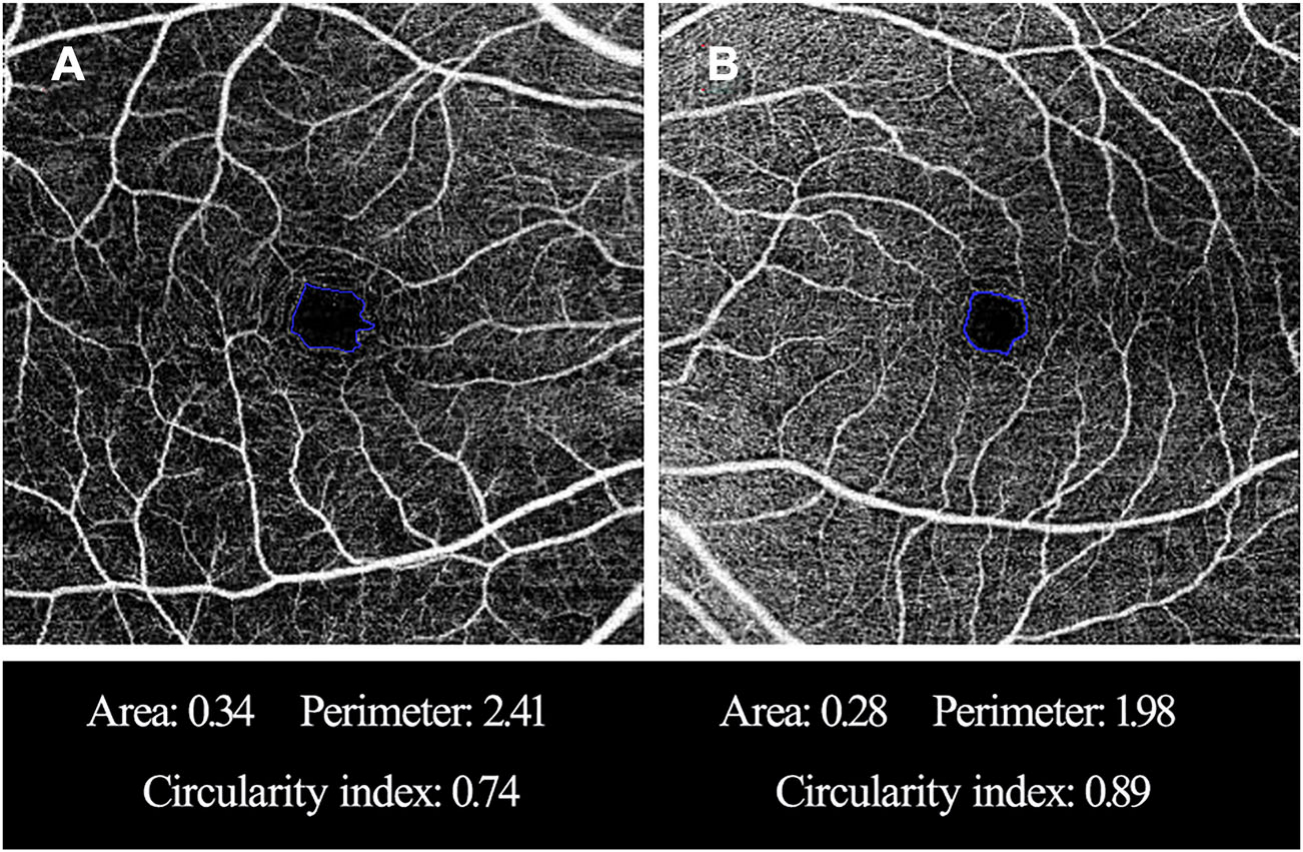
Representative cases with different foveal avascular zone (FAZ) metrics. **A** A glaucoma eye has a larger area, a longer perimeter and a lower circularity index. **B** A healthy eye has a relatively smaller FAZ area, a shorter perimeter and a higher circularity index

Our study included 48 eyes of 48 PACG patients and 31 eyes of 31healthy persons that met our initial inclusion criteria. The OCTA image quality of 10 eyes in glaucoma group was unqualified, while the OCTA image quality of 6 eyes in healthy group was unqualified. Therefore, 38 glaucomatous patients (38 eyes) and 25 healthy participants (25 eyes) were finally contained in this study. Table 1 gives the demographic and clinical characteristics of all subjects. The length of the ocular axis in glaucoma group was obviously different from that in the healthy group (*P* < 0.001). However, there was no significant difference in age, sex, IOP, and BCVA between the two groups (all *P* > 0.05). Except for SN sector, global and each regional macular SVD of the glaucoma group were lower than that of the healthy group (all *P* < 0.05).We found that the greatest reduction of vessel density in glaucomatous eyes was the macular II sector. Global and each regional macular GCIPL thicknesses of the glaucoma group were thinner than that of the healthy group (all *P* < 0.001). Compared with the control eyes, the FAZ of glaucomatous eyes had larger area, longer perimeter and lower circularity (all *P* < 0.05). Figure 2 shows typical cases.

**Table 1:** Demographic and clinical characteristics of the study population

	Healthy eyes (25 eyes)	PACG eyes (38 eyes)	*P* value
Gender (male/Female)	7:18	17:21	0.181*
Age (years)	61.6 ± 7.8	62.4 ± 8.5	0.708
BCVA	0.6 ± 0.2	0.5 ± 0.3	0.127^†^
IOP at the scanning visit (mmHg)	13.5 ± 2.7	15.0 ± 3.9	0.118
Al (mm)	23.5 ± 0.8	22.4 ± 1.1	< 0.001
VF MD	–	7.36 ± 3.36	–
Macular SVD	15.82 ± 0.99	14.82 ± 1.14	0.001
SN sector	17.05 ± 1.48	16.5 ± 1.38	0.109
SS sector	14.89 ± 1.33	14.01 ± 1.78	0.039
ST sector	16.57 ± 1.27	15.56 ± 1.28	0.003
IN sector	16.72 ± 1.09	15.69 ± 1.85	0.019^†^
II sector	14.86 ± 1.41	13.36 ± 1.79	0.001
IT sector	15.04 ± 1.39	13.80 ± 1.54	0.002
GCIPL	66.0 ± 4.8	56.0 ± 6.1	< 0.001
SN sector	76.4 ± 5.7	63.6 ± 10.2	< 0.001^†^
SS sector	71.2 ± 5.4	57.7 ± 8.8	< 0.001^†^
ST sector	72.6 ± 5.4	60.0 ± 7.1	< 0.001
IN sector	74.0 ± 5.7	60.7 ± 9.9	< 0.001^†^
II sector	66.9 ± 5.4	55.7 ± 7.1	< 0.001
IT sector	72.9 ± 5.4	59.5 ± 7.5	< 0.001
FAZ area (mm^2^)	0.35 ± 0.11	0.43 ± 0.12	0.013
FAZ perimeter (mm)	2.26 ± 0.35	2.67 ± 0.41	< 0.001
FAZ circularity index	0.85 ± 0.05	0.75 ± 0.06	< 0.001

*PACG* Primary angle closure glaucoma, *BCVA* Best-corrected visual acuity, *IOP* Intraocular pressure, *AL* Axial length, *VFMD* Visual field mean deviation, *SVD* Superficial vessel density, *SN*, Superonasal, *SS* Superosuperior, *ST* Superotemporal, *IT* Inferotemporal, II Inferoinferior, *IN* inferonasal, *GCIPL* Ganglion cell-inner plexiform layer, *FAZ* Foveal avascular zone;

All values are mean ± standard or percentages

Unless otherwise illustrated, the comparison was made by using independent t test

^*^The comparison was performed by using the Chi-square test

^**†**^The comparison was performed by using the Mann–Whitney test

### Reproducibility of FAZ measurements

The intraobserver and interobserver reproducibilities were high for FAZ measurements. The ICCs for intraobserver reliability (WC) of FAZ area, perimeter and circularity measurements were 0.993 (95% CI = 0.989–0.996), 0.984 (95% CI = 0.974–0.991), and 0.885 (95% CI = 0.811–0.930), respectively. The ICCs for interobserver reliability (WC and AZ) of FAZ area, perimeter and circularity measurements were 0.993 (95% CI = 0.988–0.996), 0.982 (95% CI = 0.970–0.989), and 0.880 (95% CI = 0.802–0.928), respectively.

### Spatial positional relationship between macular SVD and GCIPL thickness

**Fig. 3:**
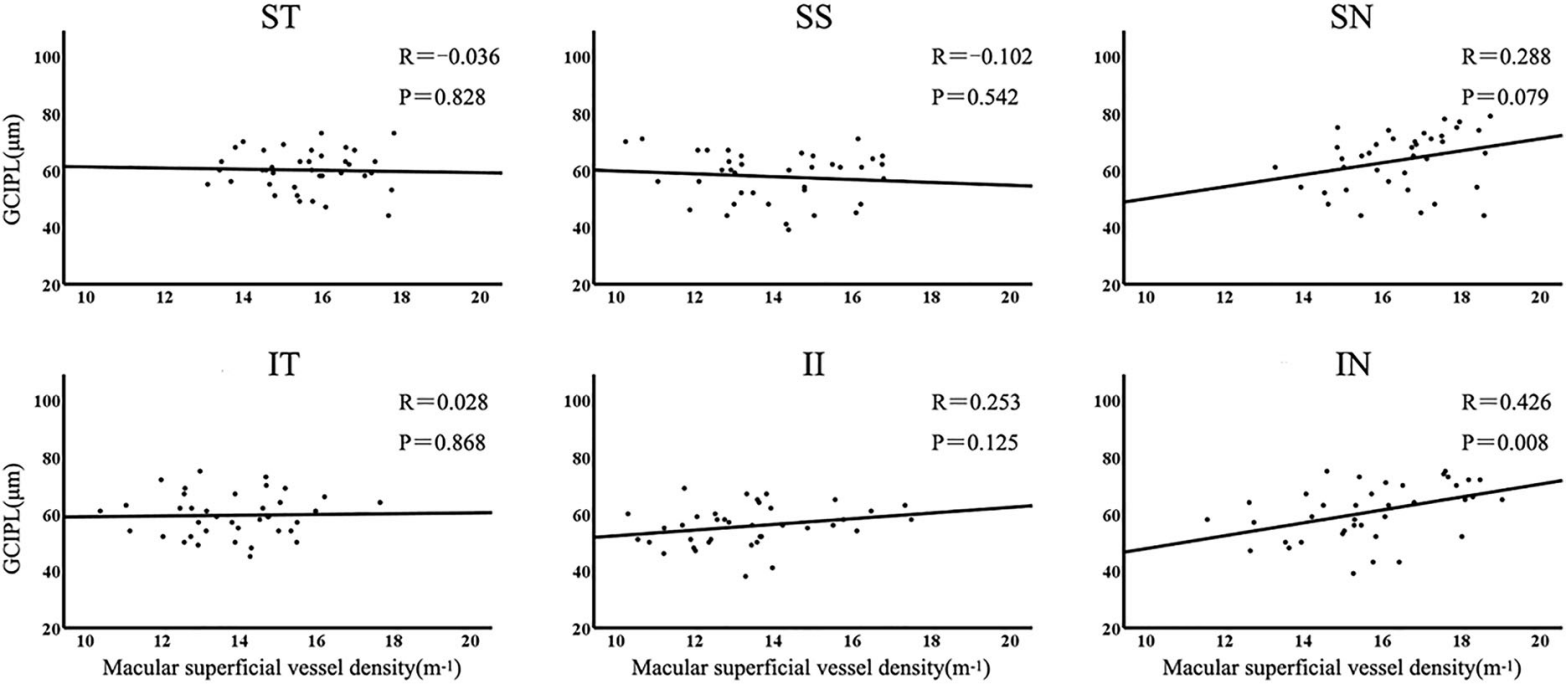
Correlation between sector macular superficial vessel density and corresponding GCIPL thickness. GCIPL, ganglion cell-inner plexiform layer; SN, superonasal; SS, superosuperior; ST, superotemporal; IT, inferotemporal; II, inferoinferior; IN, inferonasal

Macular GCIPL thickness had a moderate correlation with the macular SVD in the IN sector (r = 0.426, *P* = 0.008). There was no any statistical difference in the correlation among the other 5 sectors (Fig. 3).

### Correlation between macular SVD and pRNFL thickness

There were strong correlations between the IN sector of macular SVD and 5,6,7,8 clock-hour regions of pRNFL thickness (all r > 0.5). The II sector of macular SVD and 6,7, clock-hour regions of pRNFL thickness likewise had strong correlations (all r > 0.5). Correlations between the IN sector of macular SVD and 1,10 clock-hour regions of pRNFL thickness were considered as a random event, and the correlation of these parameters had not been reported previously (Table 2).

**Table 2:** The correlation between sector macular superficial vessel density and each clock-hour pRNFL thickness

	SN		SS		ST		IN		II		IT	
	r	*P*	r	*P*	r	*P*	r	*P*	r	*P*	r	*P*
*pRNFL*												
S	0.146	0.382	– 0.084	0.614	0.091	0.585	0.495	0.002	0.345	0.034	0.330	0.043
T	– 0.022	0.897	– 0.069	0.681	0.082	0.623	0.578	0.000	0.440	0.006	0.045	0.790
I	0.099	0.552	– 0.037	0.827	0.184	0.268	0.653	0.000	0.589	0.000	0.169	0.311
N	0.134	0.423	-0.094	0.573	0.169	0.311	0.412	0.010	0.242	0.144	0.193	0.245
1	0.241	0.144	– 0.028	0.866	0.159	0.341	0.502	0.001	0.304	0.063	0.403	0.012
2	0.270	0.101	– 0.026	0.879	0.206	0.214	0.476	0.003	0.332	0.042	0.230	0.165
3	0.006	0.973	– 0.089	0.594	0.063	0.707	0.245	0.138	0.147	0.380	0.213	0.198
4	0.065	0.698	– 0.114	0.497	0.257	0.119	0.343	0.035	0.130	0.438	0.055	0.744
5	0.252	0.126	0.033	0.845	0.061	0.715	0.515	0.001	0.397	0.014	0.219	0.186
6	0.185	0.267	0.097	0.563	0.088	0.599	0.557	0.000	0.559	0.000	0.055	0.743
7	– 0.067	0.691	– 0.161	0.336	0.173	0.300	0.536	0.001	0.570	0.000	0.088	0.601
8	– 0.124	0.460	– 0.020	0.903	0.165	0.321	0.557	0.000	0.463	0.003	-0.021	0.900
9	0.102	0.543	0.036	0.830	0.054	0.746	0.450	0.005	0.325	0.047	0.105	0.513
10	0.002	0.991	– 0.138	0.408	0.005	0.978	0.516	0.001	0.368	0.023	0.054	0.749
11	0.065	0.697	– 0.179	0.283	0.043	0.796	0.415	0.010	0.291	0.076	0.203	0.222
12	0.110	0.513	– 0.034	0.840	0.063	0.708	0.465	0.003	0.352	0.030	0.329	0.044

*SN* Superonasal, *SS* Superosuperior, *ST* Superotemporal, *IT* Inferotemporal, *II* Inferoinferior, *IN* Inferonasal, *pRNFL* Peripapillary retinal nerve fiber layer

Correlation analysis was made by using Pearson correlation test

### Diagnostic accuracies of OCT, OCTA and FAZ metrics

Tables 3 and 4 show AUCs and sensitivities at fixed specificities of macular SVD, FAZ, pRNFL thickness and macular GCIPL thickness parameters. The AUCs of macular SVD ranged between 0.61 (SN sector) and 0.76 (II sector). The AUCs of FAZ metrics ranged between 0.68 (FAZ area) and 0.94 (FAZ circularity index). The AUCs of macular GCIPL thicknesses ranged between 0.87 (SN sector) and 0.93 (ST sector). The AUCs of pRNFL thicknesses ranged between 0.62 (3 o'clock sector) and 0.92 (7 o'clock sector and inferior quadrant). The AUC was the highest for FAZ circularity index (0.94;95% CI, 0.85–0.99), followed by the ST sector of macular GCIPL thicknesses (0.93;95% CI,0.83–0.98).

**Table 3:** Diagnostic ability of pRNFL and macular GCIPL parameters (figures in parenthesis represent 95% CIs)

Optic disk	AUC	Sensitivity at 95% specificity	Sensitivity at 80% specificity
*pRNFL thickness*			
Average	0.91(0.81–0.97)	82% (66–92)	82% (66–92)
Temporal	0.82(0.70–0.91)	47% (31–64)	71% (54–85)
Superior	0.86(0.75–0.94)	74% (57–87)	76% (60–89)
Nasal	0.65(0.52–0.76)	21% (10–40)	42% (26–59)
Inferior	0.92(0.82–0.97)	84% (69–94)	87% (72–96)
9	0.72(0.59–0.82)	34% (20–54)	45% (29–62)
10	0.85(0.74–0.93)	61% (43–83)	74% (57–87)
11	0.88(0.78–0.95)	61% (44–76)	82% (66–92)
12	0.79(0.67–0.88)	47% (31–64)	63% (46–78)
1	0.85(0.74–0.93)	63% (46–78)	76% (60–89)
2	0.66(0.53–0.78)	34% (20–51)	38% (22–57)
3	0.62(0.49–0.74)	24% (11–40)	37% (22–54)
4	0.64(0.51–0.75)	34% (20–51)	47% (31–64)
5	0.83(0.71–0.91)	71% (54–85)	87% (72–96)
6	0.86(0.75–0.94)	71% (54–85)	76% (60–89)
7	0.92(0.82–0.97)	71% (54–85)	87% (72–96)
8	0.74(0.62–0.85)	29% (15–49)	50% (33–67)
*Macular GCIPL thickness*			
Average	0.91(0.81–0.97)	66% (49–87)	76% (57–90)
SN sector	0.87(0.76–0.94)	59% (39–71)	71% (54–85)
SS sector	0.92(0.83–0.98)	68% (51–85)	84% (69–94)
ST sector	0.93(0.83–0.98)	74% (57–89)	84% (69–94)
IN sector	0.88(0.77–0.95)	66% (49–83)	74% (57–87)
II sector	0.90(0.80–0.96)	68% (51–85)	79% (63–92)
IT sector	0.92(0.83–0.98)	74% (54–89)	84% (69–94)

*pRNFL* Peripapillary retinal nerve fiber layer, *GCIPL* Ganglion cell-inner plexiform layer *SN* Superonasal, *SS* Superosuperior, *ST* Superotemporal, *IT* Inferotemporal, *II* Inferoinferior, *IN* Inferonasal

**Table 4:** Diagnostic ability of macular SVD and FAZ parameters

	AUC	Sensitivity at 95% specificity	Sensitivity at 80% specificity
*Macular superficial vessel density*			
Average	0.74(0.62–0.85)	34% (20–51)	50% (33–67)
SN sector	0.61(0.48–0.73)	05% (01–18)	37% (22–54)
SS sector	0.65(0.52–0.76)	37% (22–54)	47% (31–64)
ST sector	0.71(0.58–0.82)	32% (18–51)	50% (33–67)
IN sector	0.68(0.55–0.79)	29% (15–46)	50% (33–67)
II sector	0.76(0.63–0.86)	45% (29–62)	68% (51–83)
IT sector	0.73(0.60–0.83)	42% (26–59)	45% (29–62)
*Foveal avascular zone*			
FAZ area	0.68(0.55–0.79)	21% (10–37)	47% (31–64)
FAZ perimeter	0.77(0.64–0.86)	32% (18–49)	53% (36–69)
FAZ circularity index	0.94(0.85–0.99)	66% (49–80)	95% (82–99)

*SVD* Superficial vessel density, *FAZ* Foveal avascular zone, *SN* Superonasal, *SS* Superosuperior, *ST* Superotemporal, *IT* Inferotemporal, *II* Inferoinferior, *IN* inferonasal

## Discussion

Our study found that the sector macular SVD in PACG was topographically related to corresponding GCIPL thickness only in the IN sector. In addition, pRNFL and macular SVD also showed significant correlations. The FAZ circularity index showed the highest diagnostic power.

In the current study, the macular SVD of PACG eyes was significantly lower than that of healthy eyes, which were consistent with the findings reported in earlier studies [11, 12]. Li et al. [11] and Liu et al. [12] described decrease of the macular SVD in 6 × 6 mm circular area. But they didn't investigate whether there were differences between glaucomatous eyes and healthy eyes in the sectoral division. On the contrary, we found that each sector of macular SVD in PACG eyes decreased, with the exception of SN sector, than that in the control group. The greatest reduction of vessel density in glaucomatous eyes was the macular II sector. This may be due to structural reasons. The studies of glaucoma using OCT proved that the inferior macula was more easily damaged in glaucoma [13, 27].

The macular GCIPL thickness had an important correlation with the macular SVD only in the IN sector in present study which was similar to that found in POAG [18]. Richter et al. [18]'s study of POAG showed that the macular GCIPL thickness and the macular vessel density were significantly correlated only II sector. Kim et al. [19] 's research on glaucoma-suspect and early NTG reported that macular GCIPL thickness and macular SVD had important correlation in the ST, IT and II regions. This difference may be explained by the different pathogenesis of different types of glaucoma [28]. The macular GCIPL injuries during different IOP elevation may be different. This glaucomatous injury of PACG and POAG is more closely related to IOP which is different from NTG. Depending on our current results, IN and II sectors of macular SVD showed strong topographic correlations with inferior clock-hour regions of pRNFL thickness in PACG. This may be related to the fact that most of the inferior region of the macula projects to the inferior quadrant of the disk, a region that is particularly susceptible to glaucomatous damage [13].

The FAZ is highly sensitive to ischemia. Atrophic changes of macular capillaries might first affect the shape and size of FAZ, which had been proved to have significant clinical application value in retinal vein occlusion and diabetic retinopathy [15, 16, 29]. However, few studies investigated the clinical value of FAZ in glaucoma, especially PACG. Liu et al. [12] reported that the FAZ circularity index showed high diagnostic power for detecting acute primary angle closure eyes in PACG patients after acute primary angle closure episodes. This was similar to our result. However, previous studies did not compare the diagnostic value of macular vessel density and FAZ parameters with traditional OCT measurement of pRNFL and macular GCIPL thicknesses metrics. Our study demonstrated that the diagnostic abilities of macular vessel densities were not better than the traditional pRNFL and macular GCIPL thickness measurements in PACG. However, FAZ circularity index showed the highest diagnostic power.

The advantage of the present study was that we used imageJ software to calculate the vessel density values that matched the macular GCIPL regions of each partition. In this way, each vessel density region could be accurately matched with the macular GCIPL region for correlation analysis. When to calculate the vessel density, we removed the large vessels to reduce the influence on the measurement of vessel density, because the large vessels did not participate in microcirculation perfusion. Moreover, large vessels would produce low vessel density areas around the large vessels, which might lead to false low vessel density values. Our algorithm had no such error, so it could accurately reflect the perfusion state.

The limitation was that we couldn't remove the projection artifacts of superficial vessels in deep vessels, so we couldn't investigate the correlation between deep macular vessel density and macular GCIPL thickness. Another possible limitation of current research was that most of our patients were patients with severe glaucoma. We couldn't divide into groups. Future research should evaluate the diagnostic ability of FAZ parameters for mild-to-moderate glaucoma.

Our research showed that OCTA might provide useful information for glaucoma. The important correlation between pRNFL, macular GCIPL thicknesses and macular SVD in topographic features could provide a basis for clinicians to comprehensively explain glaucoma damage. A better understanding of the correlation between macular SVD parameters and macular GCIPL, pRNFL thicknesses might broaden the application range of macular SVD. Perhaps these vascular parameters will gradually become important in the etiology, diagnosis, progression and treatment choice of glaucoma patients in the future. As a cross-sectional study, we couldn't assess whether vessel changed occur before structural changed in glaucomatous development. Future research should longitudinally evaluate the dynamic relationship between vessel changes and structural changes.

In conclusion, sector of macular SVD not only had a spatial positional correlation with corresponding macular GCIPL thickness, but also with clock-hour regional pRNFL thicknesses in PACG eyes. The SVD of macular area in glaucoma patients was sparser than that of control eyes. The FAZ circulation index might be a useful diagnostic parameter.

## Data Availability

The author confirms that all relevant data are included in the article.
